# CALB Immobilized onto Magnetic Nanoparticles for Efficient Kinetic Resolution of Racemic Secondary Alcohols: Long-Term Stability and Reusability

**DOI:** 10.3390/molecules24030490

**Published:** 2019-01-30

**Authors:** Xiu Xing, Jun-Qi Jia, Jing-Fan Zhang, Zi-Wen Zhou, Jun Li, Na Wang, Xiao-Qi Yu

**Affiliations:** Key Laboratory of Green Chemistry Technology, Ministry of Education, College of Chemistry, Sichuan University, Chengdu 610064, China; 2017222030155@stu.scu.edu.cn (X.X.); scujunqi@163.com (J.-Q.J.); zhangjingfan21@gmail.com (J.-F.Z.); 2015141231245@stu.scu.edu.cn (Z.-W.Z.); 2017222030159@stu.scu.edu.cn (J.L.)

**Keywords:** CALB, magnetic nanoparticles, cross-linked enzyme aggregates, resolution, secondary alcohols

## Abstract

In this study, an immobilization strategy for magnetic cross-linking enzyme aggregates of lipase B from *Candida antarctica* (CALB) was developed and investigated. Magnetic particles were prepared by conventional co-precipitation. The magnetic nanoparticles were modified with 3-aminopropyltriethoxysilane (APTES) to obtain surface amino-functionalized magnetic nanoparticles (APTES–Fe_3_O_4_) as immobilization materials. Glutaraldehyde was used as a crosslinker to covalently bind CALB to APTES–Fe_3_O_4_. The optimal conditions of immobilization of lipase and resolution of racemic 1-phenylethanol were investigated. Under optimal conditions, esters could be obtained with conversion of 50%, enantiomeric excess of product (ee_p_) > 99%, enantiomeric excess of substrate (ee_s_) > 99%, and enantiomeric ratio (E) > 1000. The magnetic CALB CLEAs were successfully used for enzymatic kinetic resolution of fifteen secondary alcohols. Compared with Novozym 435, the magnetic CALB CLEAs exhibited a better enantioselectivity for most substrates. The conversion was still greater than 49% after the magnetic CALB CLEAs had been reused 10 times in a 48 h reaction cycle; both ee_s_ and ee_p_ were close to 99%. Furthermore, there was little decrease in catalytic activity and enantioselectivity after being stored at −20 °C for 90 days.

## 1. Introduction

Biocatalysts have attracted more attention over the past few decades due to their broad substrate specificity, excellent selectivity, mild reaction conditions, environmental friendliness, and so on. Especially for enzymatic resolution, they provide an effective and facile route for the preparation of chiral compounds using lipase as a catalyst [1,2].

Lipase B from *Candida antarctica* (CALB) is one of the most popular and widely used lipases [3]. Its remarkable ability to catalyze a wide range of reactions, such as ammonolysis, transesterification, and esterification, is due to its excellent chemo-, regio-, and stereoselective properties [4,5,6,7]. However, like every other free enzyme, it is associated with several drawbacks, such as low stability, difficulty in product separation, and in recycling [8]. Immobilization is one of the important and efficient ways to overcome these disadvantages [1,9,10,11,12].

Immobilization of enzymes is a technology that locates enzymes onto a specific matrix support or matrix. It can improve enzyme properties, such as stability, activity, and selectivity. The carrier of an immobilized enzyme may allow complete dispersion of the enzyme molecule without possible aggregation in general. The covalent attachment between the enzyme and the carrier promotes the rigidity of the enzyme structure, which conserves the enzymatic properties under extreme conditions and reduces enzyme inhibition, thereby increasing the stability of the enzyme and conserving the enzyme activity. If medium, effector, or support are controlled in a “reasonable” manner, the enzyme will condense a structure through immobilization with better selectivity or activity, thus improving the economic viability of the operation [13,14].

Basically, methods of enzyme immobilization can be classified into three categories: binding to a support (carrier), entrapment (encapsulation), and cross-linking [1,15,16]. Binding to a support (carrier) can be physical (such as hydrophobic and van der Waals interactions), ionic, or covalent in nature. Entrapment into either a polymer network includes usually organic or inorganic polymer matrices (such as polyacrylamide or silica sol–gel), or a membrane device such as hollow fibers or microcapsules. In our previous work, enzymes were immobilized on resin and entrapped in micro-emulsion-based organogels (MBGs) [17,18]. Different enzyme immobilization approaches have been reported. Mehrasbi et al. immobilized CALB on core-shell magnetic nanoparticles for production of biodiesel from waste cooking oil [19]. Nicolas et al. immobilized CALB on lysine-modified magnetic nanoparticles [20]. Zhang et al. immobilized CALB in ZnO nanowires introduced into microporous SiO2 for resolution of 1-phenylethanol [2]. In these reports, catalytic activity was improved, yet storage stability and recycling stability was still not ideal. Besides, these immobilized enzymes and the reaction system cannot be quickly and conveniently separated after the reaction, which limits the widespread usage of immobilized enzymes in the industry. Therefore, it is of great significance to develop a convenient and stable immobilized enzyme.

Cross-linked enzyme aggregates (CLEAs) are simple in preparation and wide in application and can effectively improve enzyme stability. CLEAs are carrierless macroparticles prepared by a bifunctional reagent. In fact, cross-linking mainly involves the reaction of the amino group of the lysine residues on the external surface of the enzyme. If the enzyme contains a small amount of lysine residues on its surface, the cross-linking effect will be poor. Coprecipitation of an enzyme with a polymer containing numerous free amino groups has been successfully used to overcome this problem, e.g., poly-l-lysine, polyethylene imine, or a second protein containing multiple lysine residues, such as bovine serum albumin (BSA) as a so-called “proteic feeder” [1]. CLEAs technology combines purification and immobilization into a single unit operation. However, during industrial applications of CLEAs in reactor configurations, it might be difficult to separate CLEAs from the reaction mixture for reusing. Besides, CLEAs are considered too soft, which holds back their applications [21]. Therefore, CLEAs are often combined with other carriers to obtain better mechanical stability [1,22,23,24,25]. Combining magnetic nanoparticles with strong magnetic responses, large specific surface area, and biocompatibility with CLEAs is a novel and excellent immobilization method [26,27]. The immobilized enzyme can be quickly separated from the reaction system by an external magnetic field and can therefore be recycled. Furthermore, a 3-aminopropyltriethoxysilane (APTES) functionalized magnetic carrier is a rigid multi-functional cross-linking agent that reduces the fluidity of the lipase lip and thus produces a stable open form of immobilized lipase [28,29,30].

In this context, an immobilization strategy for magnetic cross-linking enzyme aggregates was developed and investigated for CALB. Recently, we developed a magnetic cross-linked enzyme aggregate of *Thermomyces lanuginosus* lipase (TLL) activated by Tween 80, which retained 84% of its initial activity after five recycles of biotransformation. The magnetic CALB CLEAs prepared in this experiment, showed no evident decrease of the catalytic activity after ten recycles of enantiospecific reaction [31]. The purpose of this experiment is to combine enzyme immobilization systems with high enzyme loading, stable lipase open-lid conformation, and make separation and recovery more convenient. Further investigation on some properties of immobilized CALB, including optimization of immobilization conditions and stability for immobilized enzymes, are carried out. Kinetically controlled synthesis catalyzed by enzymes is affected by factors such as pH, temperature, ionic strength, solvent composition as well as enzyme and substrate properties [32]. Enantiospecific transesterification of racemic 1-phenylethanol with vinyl acetate was selected as the model reaction and was used to evaluate the catalytical efficiency of immobilized lipase; the result was then compared to free CALB and Novozym 435. The magnetic CALB CLEAs were successfully used for enzymatic kinetic resolution of fifteen secondary alcohols.

## 2. Results and Discussion

### 2.1. Chemical Characterization and Analysis

**Figure 1:**
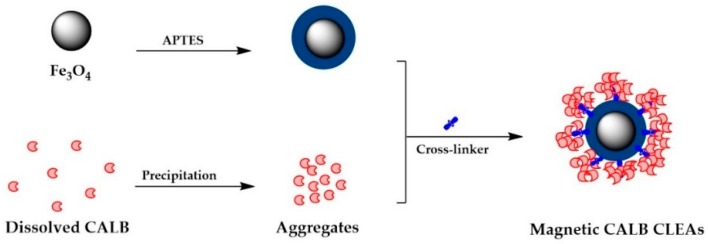
Schematic illustration of the immobilization process to produce magnetic CALB CLEAs. APTES is 3-aminopropyltriethoxysilane, CALB is lipase B from *Candida antarctica*, and CLEAs is cross-linked enzyme aggregates.

In this study, an immobilization strategy for magnetic cross-linking enzyme aggregates of CALB was developed and investigated. As illustrated in Figure 1, 3-aminopropyl triethoxysilane (APTES) functionalized magnetic nanoparticles were readily prepared and subsequently employed for the immobilization of CALB.

**Figure 2:**
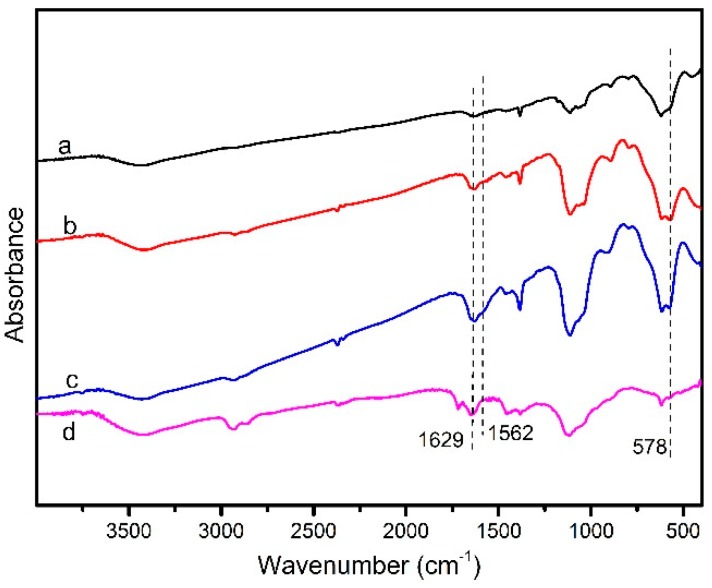
FTIR spectra of (a) Fe_3_O_4_; (b) APTES–Fe_3_O_4_; (c) magnetic CALB CLEAs; (d) CALB CLEAs.

The Fourier transform infrared spectroscopy (FTIR) spectra of Fe_3_O_4_, modified Fe_3_O_4_, magnetic CALB CLEAs, and CALB CLEAs are shown in Figure 2. A stretch in the peak at 578 cm−1 corresponded to the Fe–O vibrations of the magnetite core, and the characteristic peak that appeared indicated the structure of Fe_3_O_4_ was preserved after chemical modification (Figure 2b,c). For APTES–Fe_3_O_4_ (spectrum b in Figure 2b) the characteristic absorption peak at 1624 cm^−1^ revealed in-plane bending vibrations of CH_3_, and the peak located at 1654 cm^−1^ showed bending vibrations of NH_2_. Appearance of these characteristic IR bands confirmed that the APTES was practically incorporated and coated onto the magnetite nanoparticles. Compared with magnetic nanoparticles, the immobilized lipase displayed additional IR bands situated at 1629 cm^−1^ and 1562 cm^−1^, which represented the amide I (the stretching vibrations of C=O groups) and amide II (N–H bending and C–N stretching) bands of CALB, respectively. Spectra of the magnetic CALB CLEAs showed similar peaks of amide bands, which indicated that CALB was successfully attached to the support. The obtained peaks all matched well those in earlier reports [33].

**Figure 3:**
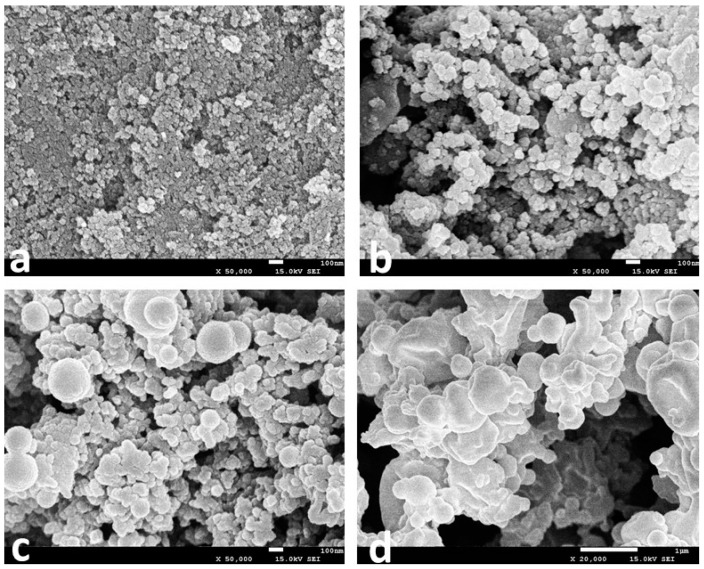
SEM images of (**a**) Fe_3_O_4_; (**b**) APTES–Fe_3_O_4_; (**c**) magnetic CALB CLEAs; (**d**) CALB CLEAs.

Figure 3 displays the scanning electron microscopy (SEM) patterns of the surface morphology of the magnetic nanoparticles and different immobilized CALB preparations. The SEM image of the bare Fe_3_O_4_ particles (Figure 3a) confirmed the particles with a size range 10–20 nm. The size of 3-aminopropyl triethoxysilane (APTES)-Fe_3_O_4_ increased to 50 nm after modification (Figure 3b). Besides, the Fe_3_O_4_ particles were arranged tightly while a looser (rough) surface structure of APTES–Fe_3_O_4_ particles was observed. This implied that the modification of APTES on the surface of the Fe_3_O_4_ nanoparticles could weaken the agglomeration of the magnetic nanoparticles and was beneficial to improving the efficiency of immobilized enzyme. When modified Fe_3_O_4_ particles were added before lipase precipitation, the magnetic particles acted as cores during lipase precipitation, whilst cross-linked with lipase. It is noteworthy that the formed APTES–Fe_3_O_4_ nanoparticles not only displayed magnetic behaviors, but also had large active surfaces available for lipase immobilization.

**Figure 4:**
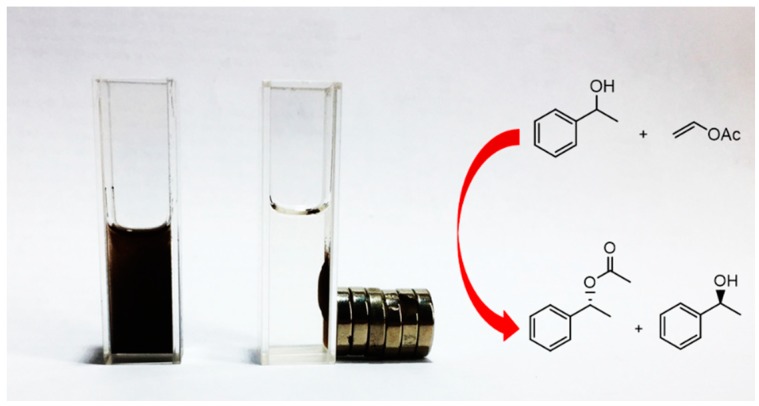
Schematic of the easy magnetic separation of magnetic CALB CLEAs.

As shown in Figure 4, all of the magnetic CLEAs showed rapid separation from the reaction mixture with the use of a magnet. The immobilized enzyme could be dispersed by removing the magnetic field and then simple shaking. This provided a simple and efficient method of separating immobilized enzymes from suspensions.

### 2.2. Optimal Conditions for the Preparation of Immobilized CALB

**Scheme 1:**
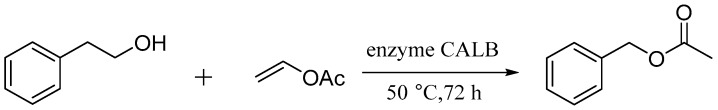
Transesterification of 2-phenylethanol and vinyl acetate.

Different immobilized conditions were investigated using transesterification of 2-phenylethanol with vinyl acetate to evaluate the activities of these biocatalysts (Scheme 1).

**Figure 5:**
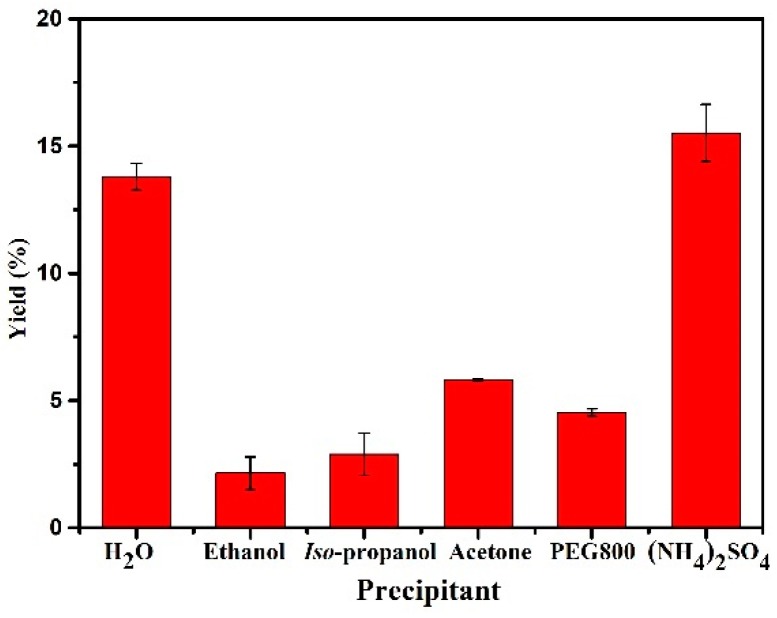
Effect of various precipitant types on the activities of magnetic CALB CLEAs. Reactions performed with 5 mg magnetic CALB CLEAs and 2 mL of n-hexane containing 0.2 mmol of 2-phenylethanol and 0.4 mmol of vinyl acetate at 50 °C for 72 h.

Firstly, precipitant is one of the most important and crucial factors that affects the activity and stability of cross-linked enzyme aggregates. The addition of precipitating agent caused the enzyme molecules to aggregate and form a supramolecular structure [34]. Different precipitants would form aggregates with different structures; it would affect the activity and stability of the immobilized enzyme if the aggregate structure was not ideally formed. In order to determine the best precipitant for CALB, five common precipitants were chosen in preparing magnetic CALB CLEAs, including ethanol, iso-propanol, acetone, PEG_800_, and saturated ammonium sulfate. As shown in Figure 5, the activities of the immobilized enzymes with different precipitants varied greatly. The immobilized enzyme exhibited the highest activity when saturated ammonium sulfate was used as a precipitant, followed by acetone and PEG_800_. In previous studies, the optimal precipitant for *Thermomyces lanuginosus* lipase was also saturated ammonium sulfate [35], while that for cellulase was isopropanol [36]. As can be seen, the best precipitants varied depending on the type of enzyme, and precipitant played a significant role in the immobilization step. Saturated ammonium sulfate was used in subsequent experiments.

**Figure 6:**
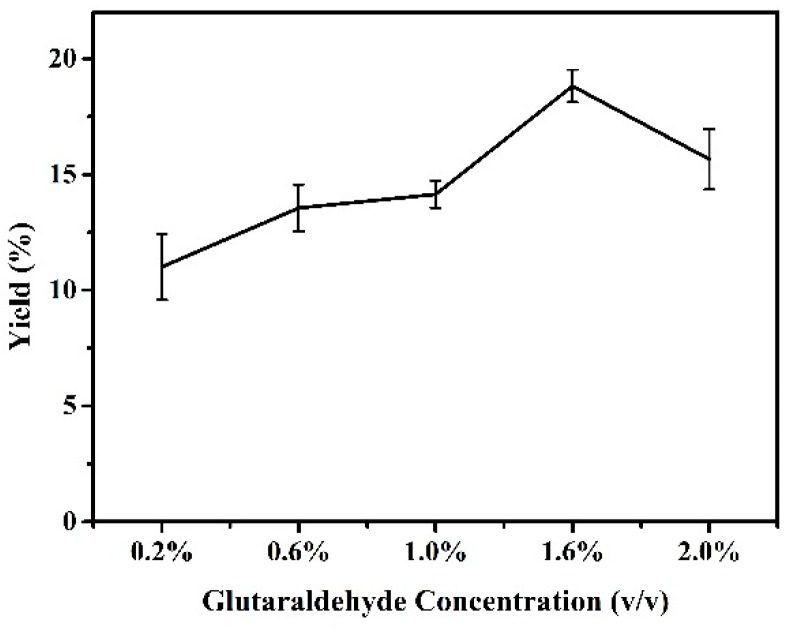
Effect of glutaraldehyde concentration on activity of magnetic CALB CLEAs. Reactions performed with 5 mg magnetic CALB CLEAs and 2 mL of n-hexane containing 0.2 mmol of 2-phenylethanol and 0.4 mmol of vinyl acetate at 50 °C for 72 h.

Cross-linking of precipitated CALB with amino-functionalized magnetic nanoparticles is the second step in the preparation of magnetic CALB CLEAs. The concentration of crosslinker is a key factor effecting the activity, stability and particle size of magnetic CALB CLEAs. Glutaraldehyde is a powerful cross-linking agent widely used in the design of biocatalysts [37]. Here, different concentrations of glutaraldehyde were introduced to obtain effective cross-linking and recycling. As illustrated in Figure 6, activity of CALB CLEAs changed with increasing glutaraldehyde concentration, which exhibited a bell-shaped curve, and the optimal activity was achieved when glutaraldehyde concentration was 1.6% *v*/*v*. At a lower cross linker concentration, cross-linking was insufficient to affect precipitation of the enzyme. However, lipase is an enzyme with high affinity for hydrophobic surfaces. When the concentration of glutaraldehyde was more than 1.6%, the glutaraldehyde dimer imparted some hydrophobicity to the surface of the carrier, which may have caused the lipase to be adsorbed before covalent binding, thereby affecting the immobilization of the enzyme [38]. This may also have been due to a higher number of enzyme-supporting bonds per enzyme molecule that lowered the catalytic efficiency [39].

**Figure 7:**
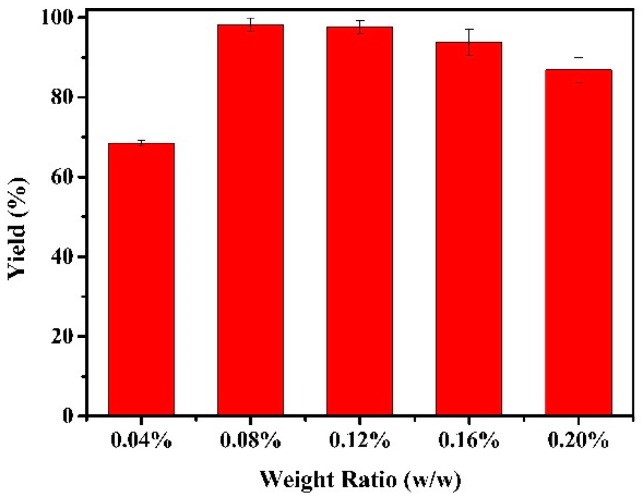
Effect of weight ratio (enzyme to nanoparticles) on activity of magnetic CALB CLEAs. Reactions performed with 5 mg magnetic CALB CLEAs and 2 mL of n-hexane containing 0.2 mmol of 2-phenylethanol and 0.4 mmol of vinyl acetate at 50 °C for 72 h.

In order to achieve effective immobilization, the transesterification yield was determined by the enzyme to nanoparticles weight ratio of 0.04% to 0.20% during the preparation of magnetic CLEAs. The immobilized CALB with ratio 0.08% (*w*/*w*) showed a significantly higher activity compared to that of 0.04% (Figure 7). Then, as the ratio gradually increased, the yield gradually decreased. Higher concentrations of enzyme may have resulted in higher enzyme loading. However, the loading efficiency decreased as the enzyme concentration increased. As shown in Figure 7, the optimal weight ratio of enzyme to nanoparticles was 0.08%.

### 2.3. Optimal Conditions for the Resolution of 1-Phenylethanol

**Scheme 2:**
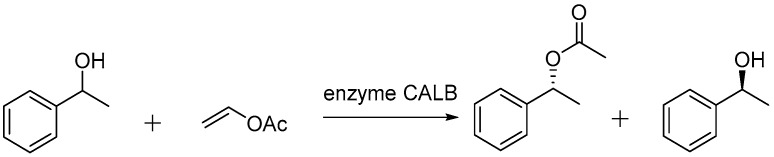
Lipase-catalyzed resolution of 1-phenylethanol.

Solvent is one of the important factors affecting the transesterification reaction, by affecting enzyme activity and substrate solubility. The effect of solvent on the resolution of 1-phenylethanol was studied (Scheme 2). As the polarity of the selected solvent decreased, both enzyme activity and selectivity increased (Table 1). It had been hypothesized that polar solvents strip away the necessary water, which binds to the enzyme by participating in non-covalent solvent protein interactions, with the result being that their active conformations were deformed [40]. On the contrary, non-polar solvents performed well, since the extreme hydrophobic nature of the solvent stabilized the hydrated enzyme structure. However, since 1-phenylethanol and vinyl acetate were both polar, once the polarity of the solvent was too low to increase the solubility of the substrate, it would be disadvantageous for the reaction. Table 1 demonstrates that with the non-polar solvent of n-hexane, the immobilized enzyme exhibited the highest activity. This was also in good agreement with results reported in the literature.

**Figure 8:**
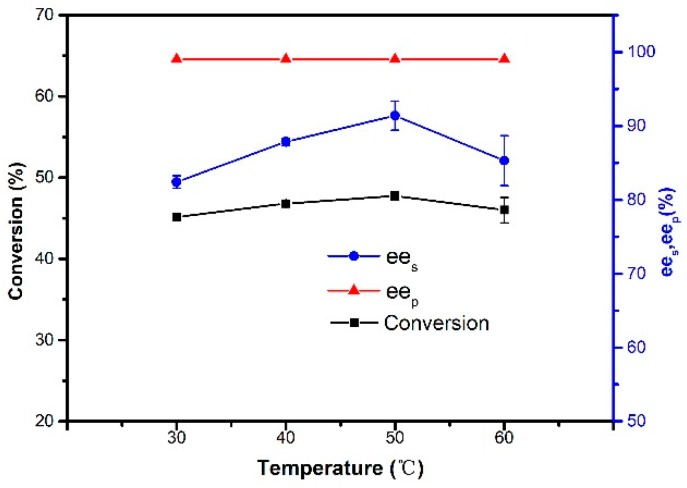
Effect of temperature on the resolution of 1-phenylethanol. Reactions performed with 5 mg magnetic CALB CLEAs and 2 mL of n-hexane containing 0.2 mmol of 1-phenylethanol and 0.4 mmol of vinyl acetate for 48 h.

The effect of reaction temperature on the resolution of 1-phenylethanol was investigated by performing the reaction with different temperatures (Figure 8). Magnetic CALB CLEAs maintained high activity and selectivity over the range 30–60 °C, and the optimum temperature was determined as 50 °C. As shown in Figure 8, the conversion and ee_s_ increased slightly as the temperature increased to 50 °C. With further temperature increases, both conversion and ee_s_ decreased slightly. It was apparent that there was no significant change in the activity of the enzyme between 30 °C and 60 °C, which indicated that immobilization could somewhat improve the thermostability of lipase. Obviously, another advantage over using biocatalysts is that the reaction can be carried out at lower temperatures, which is more energy-efficient and environmentally friendly.

**Figure 9:**
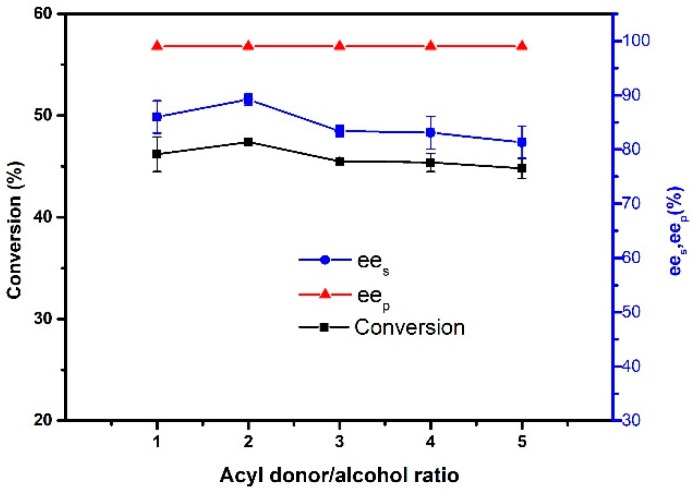
Effect of acyl donor/alcohol ratio on the resolution of 1-phenylethanol. Reactions performed with 5 mg magnetic CALB CLEAs and 2 mL of n-hexane containing 0.2 mmol of 1-phenylethanol at 50 °C for 48 h.

The vinyl acetate/alcohol molar ratio is one of the most important parameters in enzymatic transesterification reaction in this study. The reaction was investigated by changing the moles of vinyl acetate (20–100 mmol) at a constant concentration (20 mmol) of phenylethyl alcohol. It was observed that conversion increased as the molar concentration of vinyl acetate increased (Figure 9). The highest conversion and ee_s_ were obtained at a molar ratio of 2:1. It is worth noting that when the molar ratio was more than 2:1, the conversion began to decrease. This is because the target product can only be formed from the ternary complex produced by the combination of the nucleophile and reactive enzyme-substrate (acyl-enzyme or non-covalent complex) [42]. At a ratio less than 2:1, an increase in the amount of vinyl acetate caused an increase in the amount of complex. However, excessive vinyl acetate increased the polarity of the reaction medium and inactivated the enzyme in return. Therefore, the molar ratio of 2:1 was optimum for the catalytic reaction.

**Figure 10:**
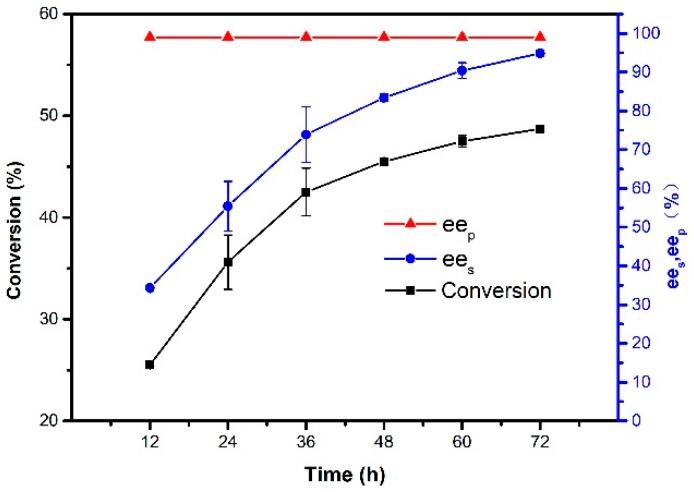
Effect of reaction time on the resolution of 1-phenylethanol. Reactions performed with 5 mg magnetic CALB CLEAs and 2 mL of n-hexane containing 0.2 mmol of 1-phenylethanol and 0.4 mmol of vinyl acetate at 50 °C.

The influence of reaction time on conversion, ee_s_, and ee_p_ was studied from 12 to 72 h at 50 °C. It can be seen from Figure 10 that the conversion increased rapidly with the extension of reaction time at the beginning, and the conversion increased slowly when reaction lasted longer than 48 h. It can be observed that the conversion and ee_s_ of immobilized lipase had already been 45% and 83% at 48 h, respectively. When reaction time was more than 48 h, the concentration of *R*-1-phenylethanol in the reaction system became very low, resulting in a slow increase in conversion. Therefore, 48 h was optimum for the reaction time.

**Figure 11:**
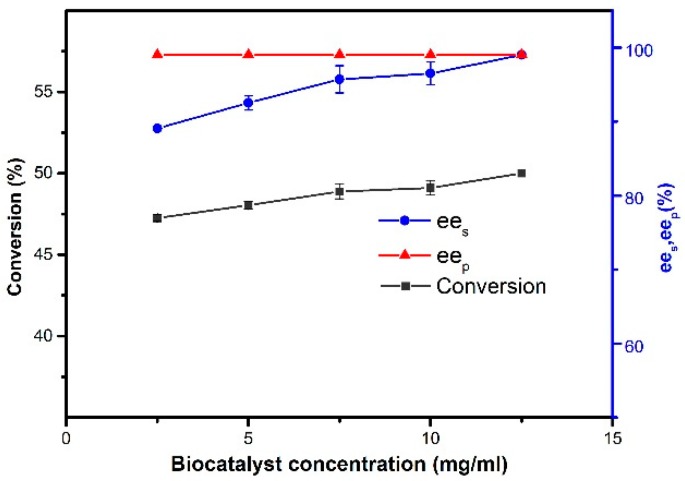
Effect of biocatalyst concentration on the resolution of 1-phenylethanol. Reactions performed with 2 mL of n-hexane containing 0.2 mmol of 1-phenylethanol and 0.4 mmol of vinyl acetate at 50 °C for 48 h.

The ratio of enzyme to substrate is also a key variable in increasing the rate of reaction and a critical feature for its potential industrial application with decisive cost. The amount of immobilized CALB added on the resolution of 1-phenylethanol was investigated from 2.5 to 12.5 mg/mL. As shown in Figure 11, as the amount of magnetic CALB CLEAs increased, both conversion and ee_s_ increased, apparently due to the relative increase in the number of enzyme active sites, which promoted the interactions between active sites and reactants. The conversion and ee_s_ were 50% and 99%, respectively, when the biocatalyst concentration was 12.5 mg/mL. In addition, there was no extreme changes in the conversion and ee_s_ at a load of 7.5 and 12.5 mg, respectively, of magnetic CALB CLEAs. This trend indicated that at high enzyme loading, the surplus enzyme particles tended to pull each other even at proper rotational motion and formed enzyme aggregates, which in turn reduced the accessibility of enzyme particles to reactants.

### 2.4. Reusability of Magnetic CALB CLEAs

**Figure 12:**
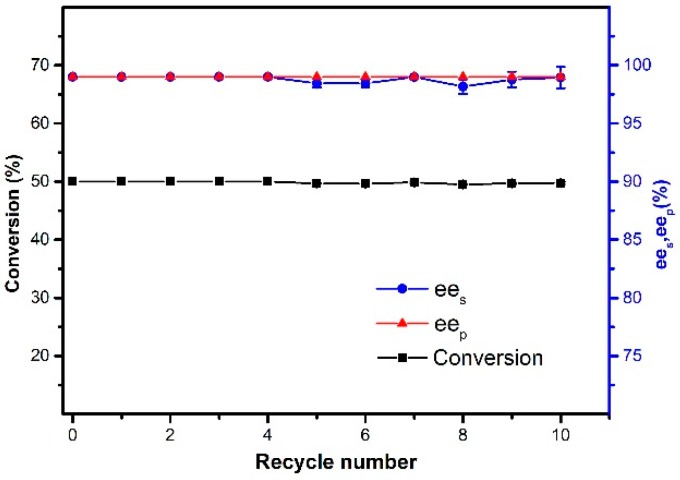
The reusability of magnetic CALB CLEAs. Reactions performed with 25 mg magnetic CALB CLEAs and 2 mL of n-hexane containing 0.2 mmol of 1-phenylethanol and 0.4 mmol of vinyl acetate at 50 °C for 48 h.

The reusability of immobilized enzyme is one of the most crucial factors that determine their suitability for practical applications. One of the advantages of magnetic nanoparticles is that they are easy to be separated and recycled, thereby increasing their economic efficiency. The reusability of the immobilized CALB was investigated under optimized conditions. After completion of the resolution reaction, the catalyst was easily separated from the reaction mixture by applying an external magnetic field. The yield of products was measured after 48 h at each cycle and the separated catalyst was washed with hexane and then dried for the next cycle. From Figure 12, it can be observed that the conversion and enantioselectivity were kept relatively constant in 10 consecutive cycles. The immobilized enzyme still afforded ca. 50% conversion with more than 98% ee_s_ and more than 99% ee_p_ in the tenth cycle.

### 2.5. Storage Stability of Magnetic CALB CLEAs

**Figure 13:**
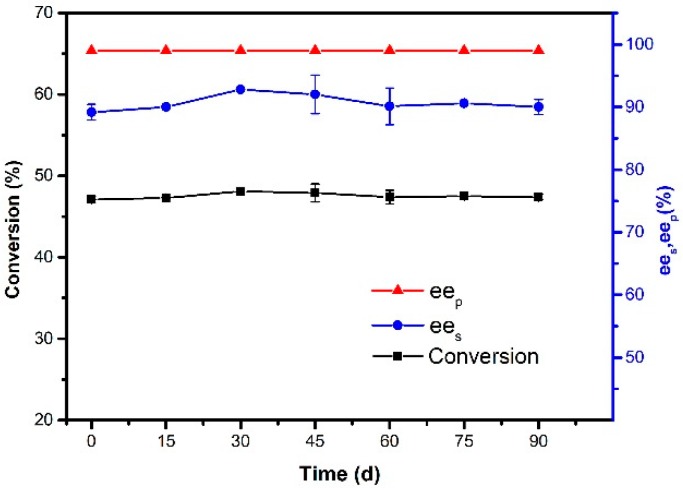
The storage stability of magnetic CALB CLEAs. Reactions performed with 5 mg magnetic CALB CLEAs and 2 mL of *n*-hexane containing 0.2 mmol of 1-phenylethanol and 0.4 mmol of vinyl acetate at 50 °C for 48 h.

The storage stability of the magnetic CALB CLEAs was tested by studying the resolution activity of the immobilized lipases at a period of time. As can been seen from Figure 13, the activity and selectivity of the enzyme were almost unchanged after storage of the immobilized enzyme at −20 °C for 90 days. The results showed that magnetic CALB CLEAs performed well in long-term storage. This result can be explained by the covalent binding of lipase on the support, thus keeping lipase stable conformation.

### 2.6. Kinetic Resolution of Different Secondary Alcohols

Encouraged by the mild optimized conditions, the kinetic resolution of fifteen secondary alcohols with vinyl acetate was conducted for broadening the application scope of the developed approach; the results are presented in Table 2. For both the magnetic CALB CLEAs and Novozym 435, the properties of substrates (e.g., dimension and electronic effects of the substituents at the stereocenter of the secondary alcohols) could influence the resolution rates. Similarly, para substituted aryl alcohols with either electron donating, entries 2 and 11, or electron withdrawing groups, entries 14–16 in Table 2, magnetic CALB CLEAs showed high activity and enantioselectivity. Apparently, the electronic properties of the phenyl substituent did not significantly affect magnetic CALB CLEAs’ activity and enantioselectivity. However, the substitution on the ortho, meta or para position altered the observed activity but did not alter the enantioselectivity. Magnetic CALB CLEAs showed the highest activity for meta-substituted alcohols, followed by ortho position. As shown in Table 2 (entry 1), the activity and selectivity of Novozym 435 and magnetic CALB CLEAs were both higher than that of free CALB. On the one hand, immobilization could avoid enzyme aggregation and inactivation. On the other hand, immobilization changed the structure of enzyme, which might be positive [13]. Noticeably, compared to Novozym 435, the magnetic CALB CLEAs exhibited better enantioselectivity towards ten alcohols, while in some other cases Novozym 435 performed better. It was because the same lipase immobilized on different supports may show different enantioselectivity [43]. Therefore, magnetic CALB CLEAs can present a promising prospect in industrial application. 

## 3. Materials and Methods

### 3.1. Materials

3-Aminopropyl triethoxysilane, glutaraldehyde (25%, *v*/*v*), 1-phenylethanol, 2-phenylethanol, and vinyl acetate were purchased from Aladdin (Shanghai, China). *E. coli* Rosetta DE3 Bovine serum albumin, Novozym 435, ampicillin, and chloramphenicol were purchased from Sigma-Aldrich (St. Louis, MO, USA). Tryptone, yeast extract, and agar were purchased from Fisher Scientific. 4-Mor-pholinepropanesulfonic acid (MOPS) (>99.5%) and Coomassie brilliant blue G250 were purchased from Sigma-Aldrich. IPTG was purchased from Shanghai Generay Biotech Co., Ltd. (Shanghai, China). All other chemicals were of analytical or chromatographical grades and used without further purification. Double distilled water was employed throughout the experiments. 

### 3.2. Expression of CALB

Single colonies of the *E. coli* Rosetta DE3 transformed cells were inoculated in a pre-culture of 5 mL Luria–Bertani (LB) media pH 7.4 containing 34 μg/L chloramphenicol and 50 μg/L ampicillin at 220 rpm 37 °C [43]. After 18 h of growth, the pre-cultures were used for inoculation of 200 mL Super broth media (32 g tryptone, 20 g yeast extract, 5 g NaCl in 1 L ddH_2_O) pH 7.4. The cultures for protein expression were incubated at 220 rpm 37 °C until optical density reached 0.6–0.8. After cooling to 16 °C, 100 μL 1mol/L isopropyl β-d-1-thiogalactopyranoside (IPTG) was added. The protein expression lasted for 18 h at 210 rpm 16 °C. The protein was harvested by osmotic shock. Prior to purification, 2 M imidazole solution was added to a final concentration of 20 mM. The protein-containing supernatant was further purified using column chromatography. Protein desalting was performed using PD 10 columns (Sephadex TM G-25 Medium columns (GE Healthcare, Uppsala, Sweden)). Final buffer was 20 mM 3-morpholinopropanesulfoinc acid (MOPS) buffer pH 7.4. The protein concentration of lipase solution was measured through the Bradford method [44].

### 3.3. Preparation of Modified Magnetic Fe_3_O_4_ Nanoparticles

Magnetic particles were prepared by the conventional co-precipitation method [26]. In a typical procedure, 0.74 g (3.7 mmol) FeCl_2_·4H_2_O and 2.02 g (7.5 mmol) FeCl_3_·6H_2_O were dissolved in 25 mL deionized water under nitrogen with vigorous stirring at room temperature. Then, as 7 mL of 30% NaOH was added drop by drop, a black precipitate formed gradually in the system. After aging for 1.5 h in the mother liquor, the resulting magnetic particles were washed several times with deionized water until neutral and dried under vacuum at −70 °C. The magnetic nanoparticles were introduced in 2.5 mL of methanol containing 25 μL of deionized water and 100 μL of APTES, and the mixture was sonicated for 30 min. Then, 1.5 mL glycerol was added, and the reaction system was refluxed at 90 °C for 6 h with mechanical agitation [36]. The particles were separated by centrifugation (10,000 rpm for 10 min) and were washed three times with methanol and water, respectively. Finally, the modified magnetic nanoparticles were dried under vacuum at −70 °C and stored at room temperature.

### 3.4. Preparation of CALB CLEAs and Magnetic CLEAs of CALB

The CALB CLEAs were prepared according to the method reported by Jia et al. [45]. Firstly, 45 mL of saturated ammonium sulphate solution was added into 5 mL of CALB solution (8 μg/mL, 20 mM MOPS buffer, pH 7.4), and stirred for 40 min at 4 °C. After precipitation of CALB, glutaraldehyde was added dropwise to the final concentration of 1.6% *v*/*v*, and stirred for 3 h at 30 °C. After cross-linking, the precipitates were separated by centrifugation at 10,000 rpm for 10 min and were washed three times with 20 mM MOPS buffer and water, respectively. Finally, the resultant precipitates were dried under vacuum at −70 °C and stored at −20 °C.

Magnetic CALB CLEAs were produced by mixing with 50 mg of APTES–Fe_3_O_4_ nanoparticles and 5 mL of CALB solution (8 μg/mL, 20 mM MOPS buffer, pH 7.4) and shaken for 20 min at 30 °C. The mixture was then ultrasonicated for 30 s. After that, 45 mL of precipitant was added with stirring at 4 °C for 40 min. After the precipitation, glutaraldehyde was added dropwise into the suspension and stirred at 30 °C for 3 h [36]. All subsequent procedures were the same as those for the CALB CLEAs preparation. 

### 3.5. Activity Assay

The yield of the ester produced by the transesterification between 2-phenylethanol and vinyl acetate was used as a reference for the enzyme activity; 5 mg of CALB-magnetic CLEAs was added to 2 mL of n-hexane containing 10mg/mL of 2-phenylethanol and 20 mg/mL of vinyl acetate. The mixture was reacted in a temperature-controlled shaker at 50 °C and 200 rpm for 24 h. The reaction was terminated by the isolation of immobilized lipase using a magnet. The samples were withdrawn from the reaction medium and analyzed by high-performance liquid chromatography (HPLC). HPLC was conducted with Waters Associates equipment (Waters 2695 with 2998 Photodiode Array Detector). A C18 column was used in the HPLC experiments with MeOH/water = 70:30 (*v*/*v*). The wavelength of the UV detector was set at 254 nm; the retention time of the product was 3.88 min. The column temperature was maintained at 30 °C during the assays, and the flow rate was 1.0 mL/min. All experiments were repeated at least three times. 

### 3.6. Calculation

The enantioselectivity value (*E*) was calculated from the conversion (*C*) and the enantiomeric excess of the product (*ee_p_*) or of the remaining substrate (*ee_s_*), based on the following equations:

(1) $$ee_{s}=\frac{S-R}{R+S}$$

(2) $$ee_{p}=\frac{S-R}{R+S}$$

(3) $$C=\frac{ee_{s}}{ee_{s}+ee_{p}}$$

(4) $$E=\frac{In\left[1-c\left(1+ee_{p}\right)\right]}{In\left[1-c\left(1-ee_{p}\right)\right]}$$

### 3.7. Characterization

#### 3.7.1. FTIR

The FTIR spectra were recorded on samples in KBr pellets using a Shimadzu FTIR-4200 spectrometer in the frequency range of 400 to 4000 cm^−1^. The samples were mixed with 1% (*w*/*w*) KBr, and the analysis was performed at 10 scans per second with a resolution of 4 cm^−1^.

#### 3.7.2. SEM

The morphology and size of the particles were observed in a scanning electron microscope (SEM, JSM 7500F, JEOL, Tokyo, Japan). The samples were freeze dried and coated with gold prior to analysis. The scanning electron microscope has a resolution of 1.0 nm and an acceleration voltage of 15.0 kV.

#### 3.7.3. HPLC

Quantitative analysis of the samples was performed via HPLC through a CHIRALPAK column (Chiral OD-3, 3μm, 4.6 mm × 250 mm; Daicel Chemical, shanghai, China) using a Shimadzu LC-2010A HT apparatus equipped with a 220 nm UV detector. Hexane with 1% (*v*/*v*) isopropanol was employed as the mobile phase with a split flow rate of 1.0 mL/min. Retention times were as follows: (*R*)-1-phenylethyl acetate, 4.78 min; (*S*)-1-phenylethyl acetate, 5.10 min; (*R*)-1-phenylethano, 15.57 min; and (*S*)-1-phenylethano, 20.23 min. The products were quantified by external standard method.

#### 3.7.4. ^1^H-NMR

^1^H-NMR spectra were obtained by a Bruker AM400 NMR spectrometer (400 MHz). Unless otherwise stated, chemical shifts (ppm) were recorded with respect to TMS in CDCl_3_. Multiplicities were defined as: s (singlet), bs (broad singlet), d(doublet), t (triplet), dd (doublet, doublet), or m (multiplet). The number of protons (n) for a given resonance is indicated by nH. Coupling constants are reported as a J values in hertz.

### 3.8. Optimization for Magnetic CALB CLEAs Preparation

Ethanol, iso-propanol, acetone, PEG_800_ (100% *w*/*v*), and a saturated ammonium sulfate solution were used to precipitate CALB as a precipitant. Furthermore, the preparation of magnetic CALB CLEAs was also done without any precipitant for reference. To obtain the optimal concentration of cross-linker, varying final concentration of glutaraldehyde in the range of 0.2%–2.0% *v*/*v* were used. Subsequently, varied concentrations of CALB were added with the constant MNPs while cross-linking; the ideal weight ratios from 0.04% to 0.20% (*w*/*w*) of free CALB and nanoparticles were then determined in order to get the maximum activity. The activities of magnetic CALB CLEAs were determined by a transesterification reaction between 2-phenylethanol and vinyl acetate.

### 3.9. 1-Phenylethanol Resolution Reusability

To analyze the reusability in application, the immobilized CALB was recycled in 1-phenylethanol resolution reaction for 10 times. The reaction was carried out at 50 °C for 48 h with constant stirring. After completion of each cycle, the immobilized CALB was separated by a permanent magnet and washed 3 times with hexane. Subsequently, the immobilized CALB was resuspended in fresh reaction mixture to begin another cycle of resolution reaction.

## 4. Conclusions

In this paper, an immobilization strategy for magnetic cross-linking enzyme aggregates was developed and investigated for CALB. Examination on some properties of immobilized CALB, included optimization of immobilization conditions and stability of immobilized enzymes. Enantiospecific transesterification of racemic 1-phenylethanol with vinyl acetate was selected as the model reaction and was used to evaluate the catalytical efficiency of immobilized lipase, which was compared to free CALB and Novozym 435. After the magnetic CALB CLEAs had been reused 10 times in a 48 h reaction cycle, the conversion was still greater than 49%, and ee_s_ and ee_p_ were close to 99%. There was also little decrease in catalytic activity and enantioselectivity after storage at −20 °C for 90 days. The magnetic CALB CLEAs was successfully used for enzymatic kinetic resolution of fifteen secondary alcohols. Compared to Novozym 435, the magnetic CALB CLEAs exhibited a better enantioselectivity for most substrates. All experiments were repeated at least three times. The magnetic CALB CLEAs exhibit excellent reproducibility in terms of activity. Therefore, magnetic CALB CLEAs can present a promising prospect in industrial application.

The NMR of compounds, chromatogram of kinetic resolution, and HPLC conditions can be found in the Supplementary Materials.

## Figures and Tables

**Table 1:** Effect of solvent on the resolution of 1-phenylethanol ^a^.

Solvents	Log P ^b^	ee_p_%	ee_s_%	C%	E
1,4-Dioxane	−1.1	38	3	5.9	2
Acetonitrile	−0.33	>99	3	3.2	206
Isopropanol	0.25	>99	1.4	1.4	202
Tetrahydrofuran	0.46	>99	5.4	5.2	210
Methyl tert-butyl ether	0.96	>99	42	29.9	300
Isopropyl ether	1.9	>99	46	31.8	316
Toluene	2.5	>99	56	36.2	351
Hexane	3.5	>99	88	47.1	587
Heptane	4.0	>99	85	46	546
Isooctane	4.5	>99	78	44.0	478

^a^ Reactions performed with 5 mg magnetic CALB CLEAs and 2 mL of solvent containing 0.2 mmol of 1-phenylethanol and 0.4 mmol of vinyl acetate at 50 °C for 48 h. ^b^ Log P values were cited from Laane et al. [41].

**Table 2:** Comparison of resolution of secondary alcohols between Magnetic CALB CLEAs and Novozym 435 ^a^.

Entry	Substrate	Catalyst	ee_p_^b^(%)	ee_s_^b^(%)	Conversion ^b^ (%)	E
1	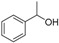	Magnetic CALB CLEAs	>99	>99	50.0	>1000
		Novozym 435	96	>99	51.1	175
		Free CALB	>99	2	2.1	204
		Fe_3_O_4_	>99	1	1.3	203
		APTES–Fe_3_O_4_	>99	2	1.9	204
2	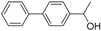	Magnetic CALB CLEAs	>99	94	48.4	>1000
		Novozym 435	94	98	50.9	149
3	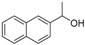	Magnetic CALB CLEAs	95	43	31.0	56
		Novozym 435	35	74.	68.1	4
4	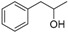	Magnetic CALB CLEAs	94	28	22.6	43
		Novozym 435	30	98	76.3	7
5	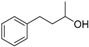	Magnetic CALB CLEAs	>99	>99	50.0	>1000
		Novozym 435	8	>99	92.1	4
6	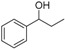	Magnetic CALB CLEAs	95	7	7.1	39
		Novozym 435	97	63	39.2	128
7	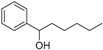	Magnetic CALB CLEAs	/	0	0	/
		Novozym 435	>99	<1	0.6	200
8	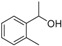	Magnetic CALB CLEAs	92	22	19.4	31
		Novozym 435	91	>99	52.1	109
9	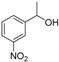	Magnetic CALB CLEAs	>99	83	45.5	605
		Novozym 435	95	>99	51.0	207
10	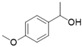	Magnetic CALB CLEAs	>99	>99	50.0	>1000
		Novozym 435	94	>99	51.4	160
11	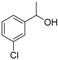	Magnetic CALB CLEAs	>99	78	44.0	472
		Novozym 435	>99	>99	50.0	>1000
12	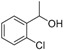	Magnetic CALB CLEAs	>99	25	20.1	254
		Novozym 435	>99	>99	50.0	>1000
13	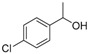	Magnetic CALB CLEAs	>99	>99	50.0	>1000
		Novozym 435	>99	>99	49.8	>1000
14	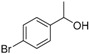	Magnetic CALB CLEAs	>99	>99	50.0	>1000
		Novozym 435	97	>99	50.7	374
15	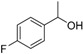	Magnetic CALB CLEAs	>99	>99	50.0	>1000
		Novozym 435	95	2.8	2.8	45

^a^ Reaction conditions: time 48 h, temperature 50 °C, 2 mL of n-hexane containing 0.2 mmol of alcohols and 0.4 mmol of vinyl acetate, and 25 mg Magnetic CALB CLEAs or 2 mg Novozym 435. ^b^ Determined by chiral HPLC analysis.

## Supplementary Materials

The Supplementary Materials are available online.
