# Supplementary material for: CALB Immobilized onto Magnetic Nanoparticles for Efficient Kinetic Resolution of Racemic Secondary Alcohols: Long-Term Stability and Reusability
Source: Molecules. 2019 Jan 30;24(3):490. doi: 10.3390/molecules24030490 (PMC6384578; doi:10.3390/molecules24030490)
Supplement: Supplementary file 1 [file molecules-24-00490-s001.pdf]

Supporting Information

Candida antarctica lipase B immobilized onto magnetic nanoparticles for efficient kinetic resolution：Long-term stability and reusability

Xiu Xing, Jun-Qi Jia, Jing-Fan Zhang, Zi-Wen Zhou, Jun Li, Na Wang*, and Xiao-Qi Yu *

Key Laboratory of Green Chemistry Technology, Ministry of Education, College of Chemistry, Sichuan University, Chengdu 610064, PR China; scujunqi@163.com
Correspondence: wnchem@scu.edu.cn, xqyu@scu.edu.cn; Tel.: +86-288-541-5886

Contents

1. HPLC conditions and NMR of various compounds………….. S1~ S6
2. References S6

1. HPLC conditions and NMR of various compounds

1.1 HPLC conditions of various secondary alcohols

Alcohol 1: 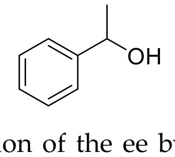

Determination of the ee by HPLC analysis: Chiral OD-3, 30 °C, n-hexane/2-propanol (99:1), 1.0 mL/min; UV 220nm; R-form: 15.6min; S-form: 20.2min.

Alcohol 2: 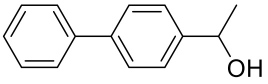

Determination of the ee by HPLC analysis: Chiral AD-H, 30 °C, n-hexane/2-propanol (99:1), 0.8 mL/min; UV 220nm; R-form: 29.4min; S-form: 33.4min.

Alcohol 3: 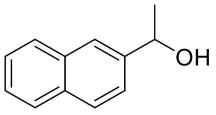

Determination of the ee by HPLC analysis: Chiral OJ, 30 °C, n-hexane/2-propanol (99:5), 1.0

mL/min; UV 220nm; R-form: 19.1min; S-form: 25.5min.

Alcohol 4: 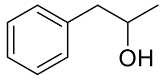

Determination of the ee by HPLC analysis: Chiral OD-3, 30 °C, n-hexane/2-propanol (99:1), 1.0 mL/min; UV 220nm; R-form: 11.8min; S-form: 13.5min.

Alcohol 5: 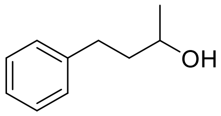

Determination of the ee by HPLC analysis: Chiral OD-3, 30 °C, n-hexane/2-propanol (99:1), 1.0 mL/min; UV 220nm; R-form: 23.1min; S-form: 38.0min.

Alcohol 6: 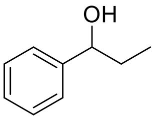

Determination of the ee by HPLC analysis: Chiral OD-3, 30 °C, n-hexane/2-propanol (99:1), 1.0 mL/min; UV 220nm; R-form: 15.6min; S-form: 20.2min.

Alcohol 7: 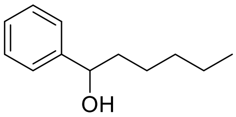

Determination of the ee by HPLC analysis: Chiral OD-3, 30 °C, n-hexane/2-propanol (99:1), 1.0 mL/min; UV 220nm; R-form: 12.8min; S-form: 14.2min

Alcohol 8: 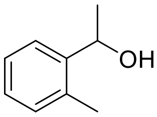

Determination of the ee by HPLC analysis: Chiral AD-H, 30 °C, n-hexane/2-propanol (99:1), 0.8 mL/min; UV 220nm; R-form: 15.5min; S-form: 17.6min.

Alcohol 9: 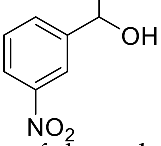

Determination of the ee by HPLC analysis: Chiral OD-3, 30 °C, n-hexane/2-propanol (99:1), 1.0 mL/min; UV 220nm; R-form: 34.1min; S-form: 37.2min.

Alcohol 10: 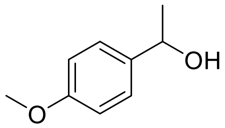

Determination of the ee by HPLC analysis: Chiral OD-3, 30 °C, n-hexane/2-propanol (99:1), 1.0 mL/min; UV 220nm; R-form: 24.2min; S-form: 27.5min.

Alcohol 11: 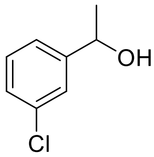

Determination of the ee by HPLC analysis: Chiral OJ, 30 °C, n-hexane/2-propanol (99:1), 1.0

mL/min; UV 220nm; R-form: 17.3min; S-form: 20.6min.

Alcohol 12: 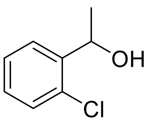

Determination of the ee by HPLC analysis: Chiral OD-3, 30 °C, n-hexane/2-propanol (99:1), 1.0 mL/min; UV 220nm; R-form: 12.6min; S-form: 13.7min.

Alcohol 13: 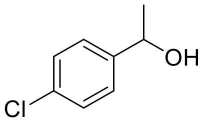

Determination of the ee by HPLC analysis: Chiral OD-3, 30 °C, n-hexane/2-propanol (99:1), 1.0 mL/min; UV 220nm; R-form: 15.6min; S-form: 17.3min.

Alcohol 14: 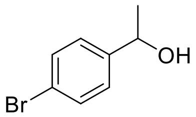

Determination of the ee by HPLC analysis: Chiral OD-3, 30 °C, n-hexane/2-propanol (99:1), 1.0 mL/min; UV 220nm; R-form: 18.1min; S-form: 20.0min.

Alcohol 15: 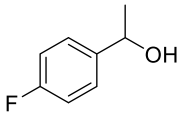

Determination of the ee by HPLC analysis: Chiral OD-3, 30 °C, n-hexane/2-propanol (99:1), 1.0 mL/min; UV 220nm; R-form: 14.7min; S-form: 15.3min.

1.2 HPLC conditions and NMR of various products:

Acetyl ester of alcohol 1: 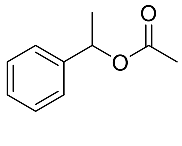

^{1}H NMR (400 MHz, cdcl$_3$) δ 7.38 – 7.27 (m, 5H), 5.88 (q, *J* = 6.6 Hz, 1H), 2.07 (s, 3H), 1.54 (d, *J* = 6.6 Hz, 3H). HPLC conditions: Chiral OD-3, 30 °C, n-hexane/2-propanol (99:1), 1.0 mL/min; R-form: 4.8min; S-form: 5.1min.

Acetyl ester of alcohol 2: 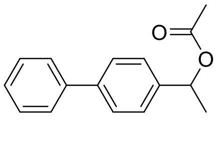

^{1}H NMR (400 MHz, cdcl$_3$) δ 7.63 – 7.55 (m, 4H), 7.49 – 7.40 (m, 4H), 7.40 – 7.31 (m, 1H), 5.93 (q, *J* = 6.6 Hz, 1H), 2.09 (s, 3H), 1.58 (d, *J* = 4.5 Hz, 3H). HPLC conditions: Chiral AD-H, 30 °C, n-hexane/2-propanol (99:1), 0.8 mL/min; UV 220nm; R-form: 7.1min; S-form: 8.6min.

Acetyl ester of alcohol 3: 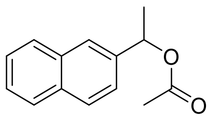

^{1}H NMR (400 MHz, cdcl$_3$) δ 7.88 – 7.78 (m, 4H), 7.52 – 7.43 (m, 3H), 6.06 (q, *J* = 6.6 Hz, 1H), 2.10 (s, 3H), 1.63 (d, *J* = 6.6 Hz, 3H). HPLC conditions: Chiral OJ, 30 °C, n-hexane/2-propanol (99:5), 1.0 mL/min; UV 220nm; R-form: 10.2min; S-form: 12.3min.

Acetyl ester of alcohol 4: 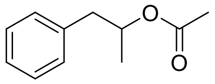

^{1}H NMR (400 MHz, cdcl$_3$) δ 7.32 – 7.17 (m, 5H), 5.11 (h, *J* = 6.4 Hz, 1H), 2.93 (dd, *J* = 13.6, 6.7 Hz, 1H), 2.75 (dd, *J* = 13.6, 6.5 Hz, 1H), 2.00 (s, 3H), 1.22 (d, *J* = 6.3 Hz, 3H). HPLC conditions: Chiral OD-3, 30 °C, n-hexane/2-propanol (99:1), 1.0 mL/min; UV 220nm; R-form: 4.9min; S-form: 6.5min.

Acetyl ester of alcohol 5: 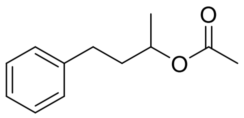

^{1}H NMR (400 MHz, cdcl$_3$) δ 7.31 – 7.25 (m, 2H), 7.22 – 7.14 (m, 3H), 4.99 – 4.89 (m, 1H), 2.72 – 2.57 (m, 2H), 2.03 (s, 3H), 1.98 – 1.89 (m, 1H), 1.85 – 1.76 (m, 1H), 1.25 (d, *J* = 6.3 Hz, 3H). HPLC conditions: Chiral OD-3, 30 °C, n-hexane/2-propanol (99:1), 1.0 mL/min; UV 220nm; R-form: 5.4min; S-form: 5.9min.

Acetyl ester of alcohol 6: 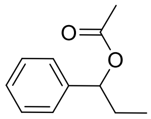

^{1}H NMR (400 MHz, cdcl$_3$) δ 7.37 – 7.30 (m, 4H), 7.30 – 7.26 (m, 1H), 5.72 – 5.62 (m, 1H), 2.08 (s, 3H), 1.93 (ddd, *J* = 14.8, 8.4, 5.4 Hz, 1H), 1.81 (ddd, *J* = 13.9, 7.4, 6.5 Hz, 1H), 0.88 (dd, *J* = 8.7, 6.1 Hz, 3H). HPLC conditions: Chiral OD-3, 30 °C, n-hexane/2-propanol (99:1), 1.0 mL/min; UV 220nm; R-form: 4.6min; S-form: 4.9min.

Acetyl ester of alcohol 7: 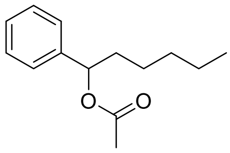

^{1}H NMR (400 MHz, cdcl$_3$) δ 7.35 – 7.28 (m, 5H), 5.72 (dd, *J* = 7.6, 6.3 Hz, 1H), 2.06 (s, 3H), 1.89 (ddt, *J* = 12.7, 9.9, 3.9 Hz, 1H), 1.79 – 1.70 (m, 1H), 1.31 – 1.24 (m, 6H), 0.88 – 0.84 (m, 3H). HPLC conditions: Chiral OD-3, 30 °C, n-hexane/2-propanol (99:1), 1.0 mL/min; UV 220nm; R-form: 4.3min.

Acetyl ester of alcohol 8: 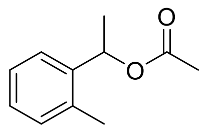

^{1}H NMR (400 MHz, cdcl$_3$) δ 7.43 – 7.37 (m, 1H), 7.25 – 7.16 (m, 2H), 7.16 – 7.12 (m, 1H), 6.08 (q, *J* = 6.6 Hz, 1H), 2.38 (s, 3H), 2.07 (s, 3H), 1.51 (d, *J* = 6.5 Hz, 3H). HPLC conditions: Chiral OD-3, 30 °C, n-hexane/2-propanol (99:1), 1.0 mL/min; UV 220nm; R-form: 4.6min; S--form:5.2min.

Acetyl ester of alcohol 9: 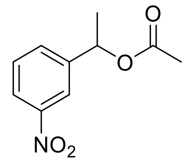

^{1}H NMR (400 MHz, cdcl$_3$) δ 7.43 – 7.37 (m, 1H), 7.25 – 7.16 (m, 2H), 7.16 – 7.12 (m, 1H), 6.08 (q, *J* = 6.6 Hz, 1H), 2.38 (s, 3H), 2.07 (s, 3H), 1.51 (d, *J* = 6.5 Hz, 3H). HPLC conditions: Chiral OD-3, 30 °C, n-hexane/2-propanol (99:1), 1.0 mL/min; UV 220nm; R-form: 8.0min; S-form: 8.5min.

Acetyl ester of alcohol 10: 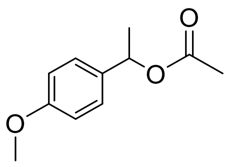

^{1}H NMR (400 MHz, cdcl$_3$) δ 7.33 – 7.27 (m, 2H), 6.90 – 6.85 (m, 2H), 5.85 (q, *J* = 6.6 Hz, 1H), 3.80 (s, 3H), 2.04 (s, 3H), 1.52 (d, *J* = 6.6 Hz, 3H). HPLC conditions: Chiral OD-3, 30 °C, n-hexane/2-propanol (99:1), 1.0 mL/min; UV 220nm; R-form: 6.0min; S-form: 6.2min.

Acetyl ester of alcohol 11: 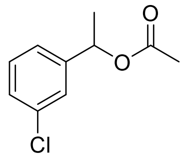

^{1}H NMR (400 MHz, cdcl$_3$) δ 7.37 – 7.18 (m, 4H), 5.83 (q, *J* = 6.6 Hz, 1H), 2.08 (s, 3H), 1.51 (d, *J* = 8.5 Hz, 3H). HPLC conditions: Chiral OJ, 30 °C, n-hexane/2-propanol (99:5), 0.8 mL/min; UV 220nm; S-form: 6.4min.

Acetyl ester of alcohol 12: 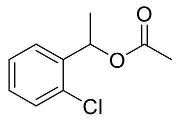

^{1}H NMR (400 MHz, cdcl$_3$) δ 7.48 – 7.41 (m, 1H), 7.37 – 7.26 (m, 2H), 7.25 – 7.17 (m, 1H), 6.21 (q, *J* = 6.5 Hz, 1H), 2.10 (s, 3H), 1.54 – 1.51 (m, 3H). HPLC conditions: Chiral OD-3, 30 °C, n-hexane/2-propanol (99:1), 1.0 mL/min; UV 220nm; R-form: 4.2min; S-form: 4.6min.

Acetyl ester of alcohol 13: 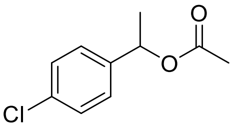

^{1}H NMR (400 MHz, cdcl$_3$) δ 7.34 – 7.26 (m, 4H), 5.83 (q, *J* = 6.6 Hz, 1H), 2.06 (s, 3H), 1.51 (d, *J* = 6.6 Hz, 3H). HPLC conditions: Chiral OD-3, 30 °C, n-hexane/2-propanol (99:1), 1.0 mL/min; UV 220nm; R-form: 4.9min; S-form: 6.5min.

Acetyl ester of alcohol 14: 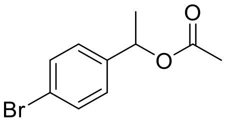

^{1}H NMR (400 MHz, cdcl$_3$) δ 7.49 – 7.45 (m, 2H), 7.24 – 7.20 (m, 2H), 5.82 (q, *J* = 6.6 Hz, 1H), 2.07 (s, 3H), 1.50 (d, *J* = 6.6 Hz, 3H). Chiral OD-3, 30 °C, n-hexane/2-propanol (99:1), 1.0 mL/min; UV 220nm; S-form:4.9min.

Acetyl ester of alcohol 15: 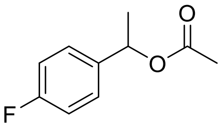

^{1}H NMR (400 MHz, cdcl$_3$) δ 7.37 – 7.27 (m, 2H), 7.07 – 6.98 (m, 2H), 5.85 (q, *J* = 6.6 Hz, 1H), 2.06 (s, 3H), 1.51 (d, *J* = 6.6 Hz, 3H). Chiral OD-3, 30 °C, n-hexane/2-propanol (99:1), 1.0 mL/min; UV 220nm; S-form: 4.6min.

References

1. Hatzakis N.S., Smonou I. Asymmetric transesterification of secondary alcohols catalyzed by feruloyl esterase from Humicola insolens. *Bioorganic Chemistry*, **2005**, 33, 325-337.
2. Chen, J.; Lin, J.H.; Xiao, J.C. Dehydroxylation of alcohols for nucleophilic substitution. *Chem. Commun.* **2018**, 54, 7034-7037.
3. Kim, H.; Choi, Y.K.; Lee, J.; Lee, E.; Park, J.; Kim, M.J. Ionic-Surfactant-Coated Burkholderia cepacia Lipase as a Highly Active and Enantioselective Catalyst for the Dynamic Kinetic Resolution of Secondary Alcohols. *Angew.Chem.* **2011**, 123, 11136-11140.
4. Chen, Q.H.; Yuan, C.Y. Synthesis of a novel ruthenium(II) complex and its unique behaviors in enzymatic dynamic kinetic resolution of secondary alcohols. *Tetrahedron* **2010**, 66, 3707–3716.
5. Lee S.Y.; Murphy J.M.; Ukai A., et al. Nonenzymatic dynamic kinetic resolution of secondary alcohols via enantioselective acylation: Synthetic and mechanistic studies. *Journal of the American Chemical Society*, **2012**, 134, 15149-53.
6. Machado, L.L.; Lemos, T.L.G.; de Mattos, M.C., et al. Immobilized Manihot esculenta preparation as a novel biocatalyst in the enantioselective acetylation of racemic alcohols. *Tetrahedron: Asymmetry* **2008**, 19, 1419–1424
7. Paivio, M.; Mavrynsky, D.; Leino, R.; Kanerva, L.T. Dynamic Kinetic Resolution of a Wide Range of Secondary Alcohols: Cooperation of Dicarbonylchlorido (pentabenzylcyclopentadienyl) ruthenium and CAL-B. *Eur. J. Org. Chem.* **2011**, 1452–1457.
